# Parental Pre-knowledge Enhances Guidance During Inquiry-Based Family Learning in a Museum Context: An Individual Differences Perspective

**DOI:** 10.3389/fpsyg.2020.01047

**Published:** 2020-06-10

**Authors:** Rooske K. Franse, Tessa J. P. Van Schijndel, Maartje E. J. Raijmakers

**Affiliations:** ^1^Department of Psychology, University of Amsterdam, Amsterdam, Netherlands; ^2^NEMO Science Museum, Amsterdam, Netherlands; ^3^Research Institute of Child Development and Education, University of Amsterdam, Amsterdam, Netherlands; ^4^Department of Education Sciences, LEARN!, Vrije Universiteit Amsterdam, Amsterdam, Netherlands

**Keywords:** *wh*-questions, parent–child interaction, pre-knowledge, individual differences, inquiry-based learning, museum context

## Abstract

Effective interaction and inquiry are an essential source for children’s learning about science in an informal context. This study investigated the effect of parental pre-knowledge on parent–child interactions (manipulations, parent talk, and child talk) during an inquiry activity in NEMO Science Museum in Amsterdam. The sample included 105 parent–child dyads (mean children’s age = 10.0 years). Half of the couples were randomly assigned to the experimental group in which, without the child’s knowledge, the parent was shown the task’s solution prior to the inquiry activity. Results show that parental pre-knowledge affected the way parents interacted and inquired with their child. Compared to parents without pre-knowledge, parents with pre-knowledge inquired longer, posed more open-ended *wh*-questions and closed questions, and less often interpreted results. Children of parents with pre-knowledge more often described evidence and interpreted results, more often manipulated alone, and solved the task more accurately. These results indicate that parental pre-knowledge brings about parents’ scaffolding behavior. In addition, it was studied how individual differences of parents and children relate to parent–child interaction. Results show that children’s self-reported inquiry attitude was related to their conversation during inquiry, such that they asked fewer closed questions and more open-ended questions. Children’s gender affected the cooperation between parent and child, parents more often manipulated together with boys than with girls, and girls more often manipulated alone. Fathers with pre-knowledge, but not mothers, let their child manipulate more by oneself than fathers without pre-knowledge. This study shows that more knowledge about an exhibit improves a parent’s scaffolding behavior in a science museum. Results are discussed in the context of museum practice.

## Introduction

Science museums offer families opportunities to learn from and about everyday science mostly by inquiry-provoking activities, which are an important means to learn about these phenomena (National Research Council, 2009). Typical for inquiry is the *gathering of evidence* by manipulating materials and observing effects, and the *interpreting of evidence* by discussing the observed effects, linking observations to pre-knowledge, and weighing the quality of gathered evidence (Schauble, 1990, 1996; Legare et al., 2017). Inquiry activities can provide parents and children with opportunities to gain insight into specific phenomena (Fender and Crowley, 2007), to develop and practice inquiry skills (Gutwill and Allen, 2010), and to experience interest in science (Crowley and Jacobs, 2002).

How parents and children inquire and what they can learn through inquiry in the museum context is of interest for developmental and educational researchers (Sobel and Jipson, 2015), as well as for museum practitioners (Allen, 2002, 2004). Parent–child interaction during inquiry activities in the museum has been studied by focusing at different aspects of behavior (Haden, 2010; Legare et al., 2017): verbal behavior (e.g., Callanan and Jipson, 2001; Gauvain, 2001; Callanan and Valle, 2008; Benjamin et al., 2010; Kisiel et al., 2012; Luce et al., 2013; Tenenbaum and Hohenstein, 2016) and non-verbal behavior (e.g., Crowley et al., 2001a; Van Schijndel et al., 2010; Willard et al., 2019) of parent, child, or parent–child dyads. Parent–child interaction during inquiry activities has been studied in different content areas, including physics (e.g., Crowley et al., 2001a), engineering (e.g., Benjamin et al., 2010), and biology (e.g., Eberbach and Crowley, 2017).

Inquiry activities offer many opportunities for learning, but does not automatically result in new knowledge or skills. Research into open-ended inquiry in the school context has demonstrated that inquiry is not always effective for concept learning and that teacher guidance (e.g., scaffolding) substantially contributes to the learning outcomes (Alfieri et al., 2011). By scaffolding, teachers temporarily bridge the gap between a learning task and children’s current abilities (Wood et al., 1976). Types of scaffolding that are used in inquiry learning are modeling, questioning, giving hints, instructing, or explaining (Van de Pol et al., 2010). Teachers can, for example, think out loud, or model how to ask questions (Rosenshine, 2009) and, by doing so, reduce the difficulty of an ill-structured task. This type of teacher support has a positive effect on children’s knowledge and skill acquisition if it is in line with the child’s pre-knowledge (Van de Pol et al., 2010; Alfieri et al., 2011). In the museum context, parents could guide their children during inquiry activities by giving individual attention and support (Crowley and Callanan, 1998; Ash, 2004; Pattison and Dierking, 2013). Research has shown that children inquire longer and on a deeper level (e.g., hypothesis-driven) if accompanied by their parents compared to inquiring alone or with peers (Gleason and Schauble, 1999; Crowley et al., 2001a). However, in general, parents are not professional teachers (Schauble et al., 2002), and it has also been demonstrated that parents can miss out on opportunities to support their children’s learning potential (Gleason and Schauble, 1999; Palmquist and Crowley, 2007; Eberbach and Crowley, 2017). For example, parents sometimes lack specific content knowledge that could enrich the verbal interaction between parent and child (Knutson and Crowley, 2010), or assume that the child’s understanding is similar to their own when interpreting evidence (Gleason and Schauble, 1999).

Parent–child interaction during inquiry activities is considered to be a collaborative and dynamic process of exploring and explaining (Legare et al., 2017). The process is described as collaborative, because both parent and child add to the learning situation by their behavior and talk, while interacting with each other (Callanan and Oakes, 1992; Gleason and Schauble, 1999). The process of parent–child interaction is described as dynamic, because the processes of *gathering evidence* through inquiry and *interpreting evidence* by drawing on prior experiences and knowledge (Siegel et al., 2007) mutually influence each other.

Within this collaborative and dynamic learning process, parent and child differences in knowledge, reasoning skills, and interest will result in opportunities for parents to scaffold their children’s learning (Wood et al., 1976; Alfieri et al., 2011). However, also for parents, content presented in the museum is sometimes new or complex. Science museums often present exhibits covering a multitude of phenomena from different content areas. Hence, it is not possible for parents to have a good understanding of all of these. This means that parents during joint inquiry often take on not only the role of facilitator of the child’s learning process but also the role of learner (Siegel et al., 2007; Falk, 2009). The present study is aimed at improving our understanding of how parents’ conceptual pre-knowledge affects parent–child interaction during inquiry activities in the museum.

### Pre-knowledge and Parent–Child Interaction During Inquiry Activities

Previous research in both formal and informal learning contexts has shown that pre-knowledge affects the way people inquire (Klahr and Dunbar, 1988; Trumbull et al., 2005) and interact (Palmquist and Crowley, 2007; Eberbach and Crowley, 2017). In a formal learning context, a lack of pre-knowledge has been shown to impede the way people experiment and make observations (Klahr and Dunbar, 1988; Trumbull et al., 2005).

In an informal learning context, two correlational studies (Palmquist and Crowley, 2007; Eberbach and Crowley, 2017), and only one experimental study (Benjamin et al., 2010) investigated the relation of *parents’*, *children’s*, and *dyads’* pre-knowledge with parent–child interaction. Eberbach and Crowley (2017) demonstrated in the context of a botanical garden, with 6- to 10-year-old children, that parental pre-knowledge is related to parent–child verbal interaction and parental guidance style. Compared to parents who knew less, parents who knew more about pollination more often talked about pollination, and more often asked their children content-related open-ended *wh*-questions during the garden visit. Open-ended *wh*-questions start with, for example, What, Why, or How, and aim at stimulating dialogue (Haden, 2010) and focusing the attention on relevant aspects for remembering and learning (Falk and Dierking, 2000; Leinhardt and Knutson, 2004; Benjamin et al., 2010). Palmquist and Crowley (2007) studied the relation of children’s pre-knowledge and parent–child interaction in the context of a Dinosaur exhibition, with 5- and 7-year-old children. It was demonstrated that children’s pre-knowledge about dinosaurs and paleontology was related to parent talk: higher amounts of talk was observed with parents of novice children compared to parents of expert children. Moreover, based on exploratory observations, children’s pre-knowledge appeared to be associated with parents’ guidance. Parents of expert children seemed “testers” of the child’s knowledge; they, for example, asked questions that encouraged children to present their knowledge about dinosaurs. Parents of novice children, on the other hand, seemed “teachers,” who supported the child’s learning process and inquired along with their child, by, for example, interpreting the information that was presented in the exhibition. These two studies showed positive relations between parents’ or children’s domain-specific pre-knowledge and parents’ domain-general guidance. However, as the studies were correlational in nature, observed differences in parental scaffolding behaviors could possibly be explained by other person characteristics than pre-knowledge. For example, parents’ attitude toward learning has shown to be related to both parental knowledge acquisition and parental guidance (Sigel, 1998; Sigel and McGillicuddy-De Lisi, 2002; Ricco and Rodriguez, 2006). Benjamin et al. (2010) investigated, in a study with an experimental design, the effect of parents’ and children’s knowledge on parent–child interaction in the context of a Building exhibition, with 4- to 8-year-old children. It was found that by receiving domain-specific knowledge prior to visiting the exhibition, parents’ and children’s content-related talk and building behavior improved: dyads’ conversations (*wh*-questions and associations) were more often domain-specific and their buildings were sturdier. Pre-knowledge, however, did not affect parental scaffolding: the total amount of *wh*-questions and associations did not increase. Possibly, the lack of effect on parental scaffolding is explained by the fact that, in this study, parent and child received the same content-related information. In the current study, we will therefore investigate the effect of parental pre-knowledge on parental scaffolding during inquiry activities in an experimental design in a museum context. We focus on 8- to 12-year-old children. Evidently, apart from parental pre-knowledge, more person characteristics of both the parent and the child are possibly relevant for parent–child interaction during inquiry, such as age (e.g., Kuhn et al., 1988; Klahr et al., 1993; Schauble, 1996), educational level (Callanan and Jipson, 2001), epistemic beliefs about learning (Ricco and Rodriguez, 2006), executive and cognitive functioning (Kirschner et al., 2006; Brigham et al., 2011; Watt et al., 2014; Zweers et al., 2019), and motivation and interest (Tomlinson et al., 2003; Vansteenkiste et al., 2004). Therefore, we will include person characteristics of parent and child in the current study. Below, we will briefly introduce research related to the impact of person characteristics on parent–child interaction in the museum context.

### Person Characteristics and Parent–Child Interaction During Inquiry Activities

Person characteristics, such as children’s and parents’ age, gender, and educational level, are reported in most museum research to give insight into the population that is studied. Some research, however, also studied how parent (e.g., Siegel et al., 2007; Tare et al., 2011; Nadelson, 2013) or child characteristics (e.g., Geerdts et al., 2015) are related to parent–child interaction. This research shows that individual differences are large, and their impact on learning and behavior in the museum might be substantial.

#### Gender

With regard to gender, it has been found that parents interact differently with boys and girls in the museum context (Crowley et al., 2001b; Siegel et al., 2007; Luce et al., 2013). Parents gave more causal explanations of science content to boys (Crowley et al., 2001b), made more absolutist statements such as claims and facts to boys (Luce et al., 2013), and behaved more collaboratively with boys (Siegel et al., 2007). Additionally, fathers and mothers have been shown to interact differently with their children in museums (Benjamin et al., 2010; Nadelson, 2013; Van Schijndel and Raijmakers, 2016). For example, father–child dyads played longer in a construction exhibition (Benjamin et al., 2010), and mothers gave more *causal* explanations (Van Schijndel and Raijmakers, 2016).

#### Interest, Motivation, and Attitude

Science interest is seen as a multi-component construct, where behavior, enjoyment, knowledge components, values, and motivational aspects mutually influence each other (Ainley, 2017; Sachisthal et al., 2018). Parents’ interest in science has been shown to be related to parent–child interaction at exhibits: parent and child engaged with more exhibits if parents had a positive science attitude (Szechter and Carey, 2009), and parent and child spent more time at exhibits if the science topic of an exhibit was of interest to parents (Tare et al., 2011). Besides science interest, other motivational aspects such as parents’ agenda to visit a museum have also been shown to be related to parent–child interaction. For example, if parents’ motivation to visit the museum was educational, parents were more involved in the child’s learning at an exhibit (Tare et al., 2011).

#### Age

One would expect children’s inquiry behaviors to be age-related (Kuhn et al., 1988; Klahr et al., 1993; Schauble, 1996). However, museum research did not show age-related differences in children’s content-related talk (Geerdts et al., 2015, 3- to 8-year-olds; Marcus et al., 2018, 4- to 8-year-olds) or manipulations (Fender and Crowley, 2007, 5- to 7-year-olds) during inquiry. In comparable age ranges, age-related differences were found in children’s conceptual understanding of the exhibit (Fender and Crowley, 2007). In addition, parents’ behavior was found to be related to children’s age (Geerdts et al., 2015; Marcus et al., 2018): compared to school-aged children, parents more often talked with preschoolers about non-observable characteristics (Geerdts et al., 2015), and science processes, technology, and engineering (Marcus et al., 2018). Other studies on parent–child conversations (Jipson and Callanan, 2003, 3- to 5-year-olds; Tenenbaum and Leaper, 2003, 11- to 13-year-olds) or manipulations (Fender and Crowley, 2007, 5- to 7-year-olds) did not find differences in parents’ behavior in relation to children’s age.

#### Educational Level

Parents’ schooling has shown to be related to how parents interact with their children in the museum context (Siegel et al., 2007; Szechter and Carey, 2009). Parents’ educational level and science museum experience was positively associated with time spent inquiring at an exhibit, and with the frequency that dyads linked the inquiry to prior experiences (Szechter and Carey, 2009). In addition, it was found that in a science museum context, higher educated parents were more directive than lower educated parents (Siegel et al., 2007).

#### Working Memory

Cognitive and executive functioning is important for learning (Kirschner et al., 2006), especially in a museum environment with a lot of distraction and open discovery tasks (Allen, 2004). An overloaded working memory can affect children’s learning experiences (Rosenshine, 2009).

#### Beliefs About Learning

Additionally, parents’ epistemological beliefs about learning might be relevant for parent–child interaction in the museum. Parents’ beliefs about learning have shown to be related to parental guidance (Sigel, 1998; Sigel and McGillicuddy-De Lisi, 2002; Ricco and Rodriguez, 2006).

### Current Study

The current study aims at a better understanding of parent–child interaction during inquiry activities, a type of activity that is at the core of the science museum experience. Parental pre-knowledge appears to play a role in parent–child interaction during inquiry, but most previous insights have stemmed from correlational research. We present a study with an experimental design, in which we manipulated parental pre-knowledge, addressing two research questions:

• How does parental pre-knowledge affect parent–child interaction during an inquiry activity in the museum? (RQ-1).
• How do person characteristics (i.e., parents’ gender, educational level, science interest and beliefs about learning, and children’s age, gender, working memory, enjoyment in science lessons, and inquiry attitude) affect parent–child interaction during an inquiry activity, and the possible relation of parental pre-knowledge and parent–child interaction? (RQ-2).

Our hypotheses are based on the idea that parents with domain-specific pre-knowledge about the phenomenon of inquiry do not have an urge for information, and therefore have the opportunity to scaffold their child’s learning process.

#### Operationalization and Hypotheses

To study the possible causal effect of parental pre-knowledge on parent–child interaction (RQ-1), a randomized controlled trial was designed, with two conditions: A control condition without pre-knowledge and an experimental condition, in which *parents* received conceptual knowledge about the phenomenon to inquire in the inquiry activity.

To be able to control *parental* pre-knowledge, a so-called black-box was used as the object of inquiry, an object that does not allow one to see from the outside what is going on inside (Miller, 2014). An important characteristic of a black-box is that no physical laws are applicable (e.g., shadow size, buoyancy, magnetism), and therefore participants cannot have pre-knowledge about the problem to be solved. This way, one can experimentally control parental pre-knowledge. The black-box used in the current study consisted of a wooden box with four holes from which four rope ends protruded. How the ropes were entangled inside the box could not be observed from the outside; however, it could be discovered by manipulating the ropes. Parents in the pre-knowledge condition were shown the entangled ropes in the inside of the box prior to the inquiry activity, without their child being aware of this disclosure. The black-box was offered in a separate room, the Research and Development lab, at the museum floor of NEMO Science Museum.

To study how person characteristics affect parent–child interaction (RQ-2), children performed a task (cognitive abilities) prior to the black-box inquiry, and parents and children filled out questionnaires afterward. Children aged between 8 and 12 participated in the study. In this age range, children can already contribute to conversations about inquiry-related topics (Chinn and Malhotra, 2002).

Parent–child interaction was measured by observing behavior and talk during inquiring at the black-box. Behavior during inquiry consisted of pulling one or more ropes, and observing what the causal effect of this manipulation was. This could be the parent’s, the child’s (solitary), or cooperative behavior. We expect that parents without pre-knowledge have an urge for information and are primarily focused on finding out for themselves how the problem can be solved. We therefore expect them to perform more manipulations of the ropes by themselves, compared to parents with pre-knowledge. We expect that parents with pre-knowledge shift from a role of learning along with their child toward taking the role of facilitating the child’s learning (Siegel et al., 2007; Falk, 2009). We therefore expect that they less often manipulate the ropes by themselves.

We quantified parents’ and children’s talk in terms of elements of scientific reasoning, such as formulating hypotheses and interpreting results, and type of explanatory talk, such as asking open-ended *wh*-questions, asking closed questions, describing evidence, and giving directions (Crowley et al., 2001a; Fender and Crowley, 2007; Gutwill and Allen, 2010; Van Schijndel and Raijmakers, 2016). We expect that pre-knowledge will affect the parental talk, such that parents with pre-knowledge will better facilitate children’s learning process. That is, we expect that parents with pre-knowledge talk more and ask more open-ended *wh*-questions to encourage children to formulate hypotheses and interpret results (Crowley and Jacobs, 2002).

## Materials and Methods

### Participants

One hundred sixteen parent–child dyads visiting NEMO Science Museum in Amsterdam participated in the study. Eleven dyads were excluded from analyses, 1 for retracting permission and 10 for technical problems. The final sample included 105 dyads consisting of an adult (P; *M*_age_ = 43.18, *SD* = 4.92; 50 male, 55 female), and a child (C; *M*_age_ = 9.96, *SD* = 1.38; 51 boys, 54 girls). To sketch a profile of the participating dyads: in all cases, the adult was the caretaker of the child. Parents were relatively highly educated (19% Graduate, 45% Bachelor, and 36% Up to Bachelor’s), and moderately interested in science. For example, half of the parents reported reading the science supplement of an (internet) newspaper weekly to monthly. Almost all parents had visited half-yearly to annual a science or natural history museum, and over half of the parents had watched or listened weekly to monthly to a science program on the radio or television. Mostly, parents believed that children learn by experimenting, reasoning, and drawing conclusions, trial and error, gaining success experiences, and receiving positive feedback from their parents (see *Section “*Measures for Person Characteristics*”*). Children were highly engaged in science, which is to be expected in a science museum.

### Procedure and Study Design

The study was conducted in the museum during spring break 2016. Families with children (8- to 12-year-olds) were approached and asked if they wanted to participate in a scientific study. If families agreed, they were welcomed in a research room where research assistants explained the procedure. The parent completed written consent forms for him- or herself and the child. Parent–child dyads were randomly assigned to a with pre-knowledge experimental condition (E, *N* = 54) or a without pre-knowledge control condition (C, *N* = 51). For both conditions, the experimental session took about 20 min and included an inquiry task that dyads could play with as long as they wanted, with a maximum of 5 min. Prior to the inquiry task and without the child being aware of it, parents in the with pre-knowledge experimental condition were shown the inside of the box. While inquiring, parent–child interaction was video recorded. When finished, parent and child were asked to make a drawing, each separately, as a measure for learning through inquiry. Furthermore, the experimental session consisted of measures to characterize the population. After the inquiry activity, parents filled out a questionnaire consisting of *Background* questions (age, gender, and educational level), *Science interest* statements, and *Beliefs about learning processes* statements. Children, prior to the inquiry task, performed a visual spatial *Working memory* task and, after inquiring, filled out a questionnaire consisting of questions about *Enjoyment in science lessons*, *Attitude to inquiry*, and *Enjoyment in science*.

### Materials

#### Inquiry Task

**FIGURE 1:**
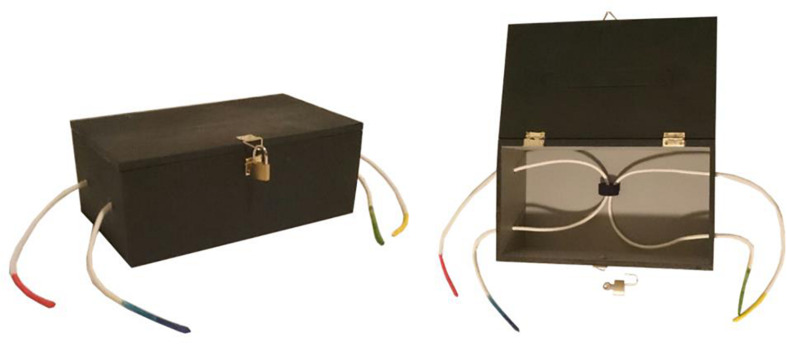
Black-box inquiry task. **(A)** Closed black-box, as presented to parent–child dyads during inquiry. Four rope ends, with unique colors (red, blue, green, and yellow) stick out. The box is sealed by a padlock. **(B)** Opened black-box, as presented to parents in the pre-knowledge experimental condition, prior to the inquiry task. Parents could observe two ropes, one with a red and blue rope end and another with a green and yellow rope end interconnected through a fabric ring.

To study the effect of parental pre-knowledge on parent–child interaction, an inquiry task was selected that, on the one hand, encouraged hypothesis-driven inquiry and, on the other hand, provided a challenge for which participants have no specific pre-knowledge, but for which pre-knowledge could be given in a quick and unambiguous way. Black-box tasks met those criteria (Lederman and Abd-El-Khalick, 1998). The black-box used in the study consisted of a wooden box (25 × 15 × 10 cm) with four holes, two ropes, one fabric ring, and a padlock (see also Figure 1). Inside the box, both ropes run through the fabric ring. Each rope end had a unique color (blue and red for rope 1, green and yellow for rope 2), and protruded through one of four box holes. When the box was closed, only the rope ends were visible, and how the ropes were entangled inside could not be observed. The way the ropes were entangled caused a complex movement pattern. This movement pattern was partly caused by the fabric ring that is not at a fixed position in the box but can also move. When someone pulled a rope, another rope end was pulled in (or multiple rope ends were pulled in). For example:

• If a participant pulls one rope (e.g., red), then the other three ropes (yellow, green, and blue) will be pulled in. How much these three ropes are pulled in depends on how tightly the participant pulls and the current position of the fabric ring in the black-box.
• If a participant pulls two ropes on the short side of the box (e.g., blue and red), then the other two ropes (green and yellow) are pulled in until the participant can’t pull the rope ends any further.
• If a participant pulls two ropes on the long side of the box (e.g., blue and green), then the other two ropes (red and yellow) are pulled in without restriction, which is that the red and yellow rope ends could disappear into the box (this, however, never happened).

Parent–child dyads were presented with the closed box and were asked to “inquire how the ropes are running on the inside.” Families were free to follow their own approach (e.g., pulling one rope at a time, or pulling two ropes simultaneously) and to inquire as long as they wanted, with a maximum of 5 min.

#### Task-Related Information (Pre-knowledge)

Parents in the experimental condition, but not in the control condition, were invited to peek into the black-box to observe how the ropes were entangled, just before the inquiry task started and without the child being aware of it.

#### Coding Approach

Parent and child’s inquiry process was recorded on video. The final scoring was based on transcripts of these recordings (in CLAN: MacWhinney and Snow, 1990). A transcript was first broken down into speech segments. A segment ended if the parent or child were taking turns or ended after a natural silence. That is, silences were included in the preceding speech segment. Parents’ and children’s manipulations during inquiry were scored by classifying each speech segment using a five-subscale coding instrument. The highest score was used for manipulations during a speech segment. In case a manipulation was continuing over multiple speech segments, it was only scored once. These subscales were: No manipulation (M_0_), Child manipulates alone (M_C_), Parent manipulates alone (M_P_), Parent and Child manipulate in parallel (M_C/P_), and Parent and Child manipulate together (M_C&P_). The inter-observer reliability for manipulations (two observers, 20% of the data) was found to be “substantial” (Landis and Koch, 1977): the percentage agreement was 85% and kappa was 0.77 (*p* < 0.001), 95% CI (0.73, 0.80). Frequencies of the five manipulation types were used as outcome variables in further analyses. Parents’ (P) and children’s (C) individual contribution to the conversations was scored by classifying each speech segment using a seven-subscale coding instrument distinguishing six different types of inquiry and guidance (Crowley et al., 2001a; Zimmerman, 2005; Fender and Crowley, 2007; Gutwill and Allen, 2010; Van Schijndel and Raijmakers, 2016). These types were as follows: Asking open-ended *wh*-questions (C1-C and C1-P for children and parents respectively; example: “Why does that rope move?”), Asking closed questions (C2-C and C2-P; example: “Is this rope attached to that rope?”), Describing evidence (C3-C and C3-P; example: “If I pull the red one then the blue one moves, but not the green one.”), Interpreting results (C4-C and C4-P; example: “The blue rope only pulls the red rope, therefore, they belong together.”), Giving direction (C5-C and C5-P; example: “Go ahead, just pull a rope.”), and Formulating hypotheses (C6-C and C6-P; example: “I expect that these four ends are actually two separate ropes.”). The seventh subscale (C7-C and C7-P) contained all unclassifiable comments such as expressing emotions (e.g., “This is really fun to do”). The inter-observer reliability for conversations (two observers, 20% of the data) was found to be “almost perfect” (Landis and Koch, 1977): the percentage agreement was 94% and kappa was 0.93 (*p* < 0.001), 95% CI (0.91, 0.95). The remaining comments category (C7-C and C7-P) was not included in the analyses. As a learning outcome variable, parents’ and children’s drawings were classified in four categories: Incorrect ropes and incorrect connection (K1), Correct ropes but incorrect fixed connection (K2), Correct ropes but incorrect loose connection (K3), and Correct ropes and correct loose connection (K4). Holding time, the number of minutes played, was used as a first explorative outcome variable to describe parent–child interaction.

To sum up, parent–child interaction is in the current study described by 17 dependent variables. That is, five manipulations variables, six parent talk variables and six child talk variables. Learning (knowledge gain) is described by four dependent variables.

#### Measures for Person Characteristics

##### Working memory (child)

Children’s visual spatial working memory was tested using the Chessboard Task (Dovis et al., 2012). This task assesses children’s ability to both maintain and manipulate visual–spatial information, and is based on the Corsi Block Tapping Task (Corsi, 1972), and the subtest Letter–Number Sequencing from the Wechsler Adult Intelligence Scale (WAIS, Wechsler, 1958). Children played for a maximum of 7 min, and on average performed 20 trials. As a measure of the child’s working memory, the longest sequence achieved during 7 min of play was reported and used in further analyses. The working memory scores are relative scores and are solely used to compare differences between parent–child dyads within the current study.

##### Enjoyment in science lessons (child) and Attitude to inquiry (child)

Subscales *Enjoyment in science lessons* (α = 0.91, 10 items) and *Attitude to inquiry* (α = 0.81, 10 items) of the Test of Science-related attitudes (TOSRA), a measure to distinguish science-related attitudes among secondary school students (Fraser, 1981), were translated to Dutch and adjusted to primary school wording. Children rated their agreement with statements on a 5-point-Likert scale (1 = strongly disagree to 5 = strongly agree). Example questions are as follows: “Science is one of the most interesting school subjects” (*Enjoyment in science lessons* sub-scale), and “I would rather solve a problem by doing an experiment than be told the answer” (*Attitude to inquiry* sub-scale). A forced two-factor analysis under Varimax rotation of the Dutch questionnaire (i.e., the translated and adjusted subscales), resulted in two process factors that contained the same items and comparable reliabilities as reported by Fraser (1981): *Enjoyment in science lessons* with α = 0.91 (cf. Fraser, α = 0.91) and *Attitude to inquiry* with α = 0.74 (cf. Fraser, α = 0.81), explaining 30 and 15% of variance, respectively. Sum scores of *Enjoyment in science lessons* and sum scores of *Attitude to inquiry* are reported and used in further analyses.

##### Additional measures for person characteristics, not used in analyses

Parents’ *Beliefs about learning processes* (i.e., learning as active or passive process) were evaluated using a 16-statement survey (How Children Learn Inventory; Ricco and Rodriguez, 2006). However, the reliabilities of the two sets of statements, α = 0.38 for learning as an active process and α = 0.45 for learning as a passive process, were insufficient to use variables based on these sets in further analyses. Explorative factor analysis resulted in one scale with 10 statements and α = 0.62, explaining 16% of the variance. In addition to children’s *Enjoyment in science lessons*, also children’s not school-related *Science enjoyment* was evaluated using a subscale of the Dutch science and technology attitude instrument for primary school pupils (Walma van der Molen et al., 2007). Children’s responses on the VTB and TOSRA subscales were found to be significantly related, *r* = 0.51, *p* < 0.001; therefore, only the results of the subscale with the highest reliability were included in the analyses (i.e., TOSRA *Enjoyment in science lessons*).

To sum up, in the analyses, seven independent variables will be used to describe parents’ and children’s person characteristics, that is, five child characteristics—age, gender, working memory, enjoyment in science lessons, and inquiry attitude—and two parent characteristics—gender and educational level.

#### Analysis Approach

To study the effect of parental pre-knowledge, person characteristics, and possible interactions between parental pre-knowledge and person characteristics on parent–child interaction, three MANCOVAs were performed, one for each aspect of parent–child interaction: manipulations, parent talk, and child talk. To further study the relationship between the dependent variables of each parent–child interaction aspect, follow-up analyses were performed using univariate ANOVAs (Field, 2009). From these analyses, we learned how the behavioral measures (e.g., the six parent talk categories) play together in different conditions (RQ-1; with and without parental pre-knowledge), or with different person characteristics (RQ-2; e.g., gender child). Levene’s Test of Equality of Error Variances was violated for six outcome variables (M_0_, M_P&C_, C1-P, C6-P, C1-C, and C2-C); therefore, we choose Hotelling’s *T* as test statistics (Field, 2009); nevertheless, robustness of the *F* statistic seems to be warranted because of equal group sizes of pre-knowledge (without = 51, with = 54), inquiry attitude (low = 55, high = 50), and gender child (boys = 51, girls = 54) groups, also see Table 2 (Blanca et al., 2018). To test the robustness of results, we performed non-parametric tests (Mann–Whitney *U* for two groups and Kruskal–Wallis for four groups) in addition to the follow-up ANOVAs, which confirmed significance of all significant ANOVA results. For the sake of brevity, these results are not reported here. With these follow-up analyses, we could describe, for example, that parental pre-knowledge led to parents asking more open-ended *wh*-questions, or that children with a higher inquiry attitude asked less closed questions.

## Results

### Descriptions of Person Characteristics and Parent–Child Interaction

#### Person Characteristics

Mean values of person characteristics used in analyses are reported in Table 1, for all participants and for participants per pre-knowledge condition. A profile description of participating dyads can be found in Participants (see “Materials and Methods” section). On average, children agreed with enjoying science lessons (*M* = 41.39, *SD* = 7.94); they scored significantly higher than the international standard (*M* = 32.8, *SD* = 9.5; Fraser, 1981), *t*(104) = 11.08, *p* < 0.001. Note that in the international standard, children were older (12- and 13-year-olds) than in the current study. Interesting is that, although they enjoyed science lessons, on average (*M* = 37.21, *SD* = 5.90) children rated themselves as having a moderate inquiry attitude (30 = not being sure of having, 40 = agreeing with having an inquiry attitude).

**TABLE 1:** Factors and covariates, describing person characteristics of parent and child.

				Parental pre-knowledge	
			Total	Without	With
**Parent**					
Gender	Male	*N* (%)	50 (48%)	22 (43%)	28 (52%)
	Female	*N* (%)	55 (52%)	29 (57%)	26 (48%)
Dyad	Father–Son	*N* (%)	26 (25%)	12 (24%)	14 (26%)
	Father–Daughter	*N* (%)	24 (23%)	10 (20%)	14 (26%)
	Mother–Son	*N* (%)	25 (24%)	13 (25%)	12 (22%)
	Mother–Daughter	*N* (%)	30 (29%)	16 (31%)	14 (26%)
Educational level	Up to Bachelor (L)	*N* (%)	38 (36%)	17 (33%)	21 (39%)
	Bachelor (B)	*N* (%)	47 (45%)	22 (43%)	25 (46%)
	Graduate (G)	*N* (%)	20 (19%)	12 (24%)	8 (15%)
**Child**					
Gender	Male	*N* (%)	51 (49%)	25 (49%)	26 (48%)
	Female	*N* (%)	54 (51%)	26 (51%)	28 (52%)
Age		*M* (*SD*)	9.96 (1.38)	9.95 (1.36)	9.97 (1.42)
Working memory		*M* (*SD*)	4.49 (0.89)	4.50 (0.87)	4.49 (0.92)
Enjoyment in science lessons		*M* (*SD*)	41.39 (7.94)	41.84 (8.05)	40.96 (7.89)
Attitude to inquiry		*M* (*SD*)	37.21 (5.90)	37.39 (7.10)	37.04 (4.56)

*Total number (*N*) and percentages (%) of the parents’ and children’s gender, the distribution of gender over the various parent–child dyads and the parents’ educational level, and average values (*M*) and standard deviations (*SD*) of the children’s age, working memory, enjoyment in science lessons and attitude to inquiry. Total = all participating parent–child dyads (*N* = 105). Without = parent–child dyads without parental pre-knowledge, control condition (*N* = 51). With = parent–child dyads with parental pre-knowledge, experimental condition (*N* = 54).*

**TABLE 2:** Outcome variables, describing parent–child interaction during an inquiry activity in the museum.

				Pre-knowledge		Inquiry Attitude		Gender Child	
Parent–child Interaction		Range *(Min–Max)*	Total *M (SD)*	Without *M (SD)*	With *M (SD)*	Low *M (SD)*	High *M (SD)*	Boys *M (SD)*	Girls *M (SD)*
**Manipulations**									
No manipulations	M_0_	5–55	24.87 (13.45)	**18.80** (11.29)	**30.59** (12.89)			22.76 (13.96)	26.85 (12.76)
Child manipulates alone	M_C_	0–30	10.53 (6.71)	**8.67** (6.59)	**12.30** (6.40)			9.39 (5.95)	11.61 (7.25)
Parent manipulates alone	M_P_	0–22	5.87 (5.19)	6.63 (5.29)	5.15 (5.04)			**5.02** (4.38)	**6.67** (5.78)
Parent and child in parallel	M_P/C_	0–14	3.23 (3.21)	3.96 (3.39)	2.54 (2.89)			3.33 (3.12)	3.13 (3.32)
Parent and child together	M_P&C_	0–16	2.89 (3.72)	2.22 (2.77)	3.52 (4.36)			**3.55** (3.89)	**2.26** (3.48)
**Parent Talk**									
Asking *wh*-questions	C1-P	0–15	3.96 (3.39)	**2.10** (2.18)	**5.72** (3.40)				
Asking closed questions	C2-P	0–19	5.35 (4.12)	**3.86** (2.94)	**6.76** (4.58)				
Describing evidence	C3-P	0–11	3.16 (2.65)	2.96 (2.49)	3.35 (2.80)				
Interpreting results	C4-P	0–19	3.65 (3.25)	**4.33** (3.39)	**3.00** (3.00)				
Giving directions	C5-P	0–24	6.69 (4.02)	5.82 (3.60)	7.50 (4.25)				
Formulating hypotheses	C6-P	0–2	0.08 (0.30)	0.08 (0.27)	0.07 (0.33)				
**Child Talk**									
Asking *wh*-questions	C1-C	0–5	0.33 (0.76)	0.41 (0.90)	0.26 (0.59)	**0.18** (0.48)	**0.50** (0.95)		
Asking closed questions	C2-C	0–5	0.75 (1.10)	0.57 (0.70)	0.93 (1.36)	**0.98** (1.13)	**0.50** (0.74)		
Describing evidence	C3-C	0–19	4.76 (4.00)	**4.00** (4.05)	**5.48** (3.86)	4.16 (3.56)	5.42 (4.38)		
Interpreting results	C4-C	0–17	6.48 (3.83)	**5.25** (3.49)	**7.63** (3.80)	6.69 (4.37)	6.24 (3.15)		
Giving directions	C5-C	0–9	1.92 (2.09)	2.00 (2.30)	1.85 (1.90)	1.80 (1.98)	2.06 (2.23)		
Formulating hypotheses	C6-C	0–3	0.14 (0.49)	0.10 (0.36)	0.19 (0.59)	0.18 (0.61)	0.10 (0.30)		

*Mean values (*M*) and standard deviations (*SD*), of three categories of outcome variables (manipulations, parent talk, and child talk), are presented for condition (parental pre-knowledge) and person characteristics (attitude to inquiry and gender child) with a main effect on parent–child interaction. Column “Total” contains range and mean values of all participating parent–child dyads (*N* = 105). Column “Without” and “With” contains values of parent–child dyads in the without parental pre-knowledge control condition (*N* = 51) and with parental pre-knowledge experimental condition (*N* = 54), respectively. Column “Low” and “High” contains values of parent–child dyads with children having a low (*N* = 55) and high (*N* = 50) attitude to inquiry, respectively. Column “Boys” and “Girls” contains values of parent–boy (*N* = 51) and parent–girl (*N* = 54) dyads, respectively. In bold: outcome variable with a significant difference between pre-knowledge conditions (without and with), inquiry attitude groups (low and high), and the child’s gender (boys and girls) respectively.*

ANOVA’s (ratio variables) and chi-square analyses (nominal variables) were performed to investigate equal distribution of the person characteristics (the parents’ gender and educational level, and the children’s gender, age, working memory, enjoyment in science lessons, and inquiry attitude) across pre-knowledge conditions. No differences between pre-knowledge conditions were found.

#### Parent–Child Interaction

Summary values, and values per pre-knowledge condition, are reported in Table 2. On average, dyads inquired the inquiry task for 3.14 min. Dyads in the with pre-knowledge experimental condition (*M* = 3.62, *SD* = 1.38), played for 1 min longer than dyads in the without pre-knowledge control condition (*M* = 2.63, *SD* = 1.52), *t*(103) = -3.483; *p* < 0.001.

When manipulating, in most cases (73%), the child (M_C_) or the parent (M_P_) manipulated alone. There was a high correlation between not manipulating the black-box and content-related talk (sum of C1–C6) of parents, *r* = 0.88, *p* < 0.001, and children, *r* = 0.67, *p* < 0.001.

Parents, *M* = 22.89, *SD* = 11.52, contributed more than children (*M* = 14.39, *SD* = 8.20), to content-related talk during inquiry, *t*(104) = 8.967, *p* < 0.001. Parents made all kinds of content-related comments, but most often gave directions to the child (C5-P), “okay, so we have to find out how the ropes are running.” Notably, they formulated almost no hypotheses (C6-P). Parents with pre-knowledge significantly contributed more to content-related talk (*M* = 26.41, *SD* = 12.06), than parents without pre-knowledge (*M* = 19.16, *SD* = 9.71), *t*(103) = 3.38, *p* < 0.001.

Children most often interpreted results (C4-C), “I think these are two different ropes,” described observations (C3-C), “all ropes have different colors,” and made non-content-related comments (C7-C), such as expressing emotions “this is really difficult!”. Children asked relatively few questions (C1-C, C2-C) and, similar to the parents, formulated almost no hypotheses (C6-C). Children of parents with pre-knowledge significantly contributed more to content-related talk (*M* = 16.33, *SD* = 7.83) than children of parents without pre-knowledge (*M* = 12.33, *SD* = 8.16), *t*(103) = 2.56, *p* < 0.05.

To give an impression of the conversations, two examples are displayed in Table 3. Hypotheses do occur (see example 1; “Yes, let’s try that. So if I pull these two, then those two go over there.”). However, sometimes participants observe the effect of a manipulation just before they fully express the hypothesis, and therefore the expression does not count as hypotheses (see in example 2; “And if we pull this, yes the yellow one goes more smoothly”).

**TABLE 3:** Two examples of parent–child conversations during inquiry at a black-box.

Example 1			
Father:	Look, that one pulls, yellow pulls, ah!	C3-P	M_P/C_
Daughter:	Now, pull that one.	C5-C	M_C_
Father:	Look at that, this one goes to the middle.	C3-P	M_C_
Daughter:	I know this one.	C7-C	M_P/C_
Daughter:	This one goes, those two.	C3-C	M_P_
Father:	Yes, and this one is.	C2-P	M_P_
Daughter:	Also connected.	C4-C	M_P_
Daughter:	Ah, I know.	C7-C	M_0_
Father:	You already know?	C2-P	M_0_
Daughter:	Ahu.	C7-C	M_0_
Father:	What do you think? Well, tell me then.	C1-P	M_0_
Daughter:	These two are together, and so are those, and they form a knot.	C4-C	M_C_
Father:	Yes, let’s try that. So if I pull these two, then those two go over there.	C6-P	M_P_
Father:	Yes, and now you pull those two. Yes, and now we pull only one.	C5-P	M_P/C_
Father:	Yes, I think you are right.	C7-P	M_0_

**Example 2**			

Father:	You pull those and then those two go in.	C3-P	M_C_
Son:	So uhm. then this one is underneath that one, I guess.	C4-C	M_0_
Son:	Pull, for example, the red one.	C5-C	M_C_
Son:	Then these two go. And if we pull this, yes the yellow one goes more smoothly.	C3-C	M_P&C_
Son:	So red goes with yellow.	C4-C	M_0_
Father:	Now pull the yellow one.	C5-P	M_C_
Father:	Then the blue one goes in.	C3-P	M_0_
Father:	Stop, stop, otherwise you’ll pull it all the way in.	C5-P	M_0_
Son:	And with this one, the yellow one goes.	C3-C	M_P/C_

*Example 1 is a full conversation of father–daughter (without pre-knowledge; 8-year-old). Example 2 is a conversation fragment of father–son (without pre-knowledge; 9-year-old). C1, asking open-ended *wh*-questions; C2, asking closed questions; C3, describing evidence; C4, interpreting results; C5, giving directions; C6, formulating hypotheses; C7, other comments. P, Parent; C, Child. M_0_, no manipulations; M_*C*_, child manipulates alone; M_*P*_, parent manipulates alone; M_*P/C*_, parent and child in parallel; M_*P&C*_, parent and child together.*

### The Impact of Parental Pre-knowledge and Person Characteristics on Parent–Child Interaction

#### Overall Results

##### Manipulations

To find out if pre-knowledge and person characteristics affected parent and child’s manipulations during inquiry, a MANCOVA was performed with the five manipulation categories (M_0_, M_C_, M_P_, M_P/C_, and M_P&C_) as outcome variables, with parental pre-knowledge, gender, and educational level, and children’s gender as factors, and with children’s age, working memory, enjoyment in science lessons and inquiry attitude as co-variates. Also, three two-way factor interactions were included (condition with respectively gender parent, educational level parent and gender child). Results showed significant main effects of parental pre-knowledge, *T* = 0.35, *F*(5,87) = 6.11, *p* < 0.001, *n*^2^ = 0.26, and children’s gender, *T* = 0.14, *F*(5,87) = 2.36, *p* = 0.05, *n*^2^ = 0.12, on manipulations. In addition, there was an interaction effect of parental pre-knowledge and parent’s gender on manipulations, *T* = 0.14, *F*(5,87) = 2.38, *p* = 0.05, *n*^2^ = 0.12.

##### Parent talk

To find out if parental pre-knowledge and parent characteristics affected parent talk, a MANOVA was performed with six conversation categories (C1-P to C6-P) as outcome variables and with parental pre-knowledge, gender and educational level as factors. Also, the two two-way factor interactions were considered (condition with respectively parent’s gender and educational level). Results showed a significant main effect of parental pre-knowledge [*T* = 0.61, *F*(6,92) = 9.36, *p* < 0.001, *n*^2^ = 0.38] on parent talk. No other significant effects were observed.

##### Child talk

To find out if parental pre-knowledge and child characteristics affected child talk, a MANCOVA was performed with six conversation categories (C1-C to C6-C) as outcome variables, with parental pre-knowledge and children’s gender as factors, and with children’s age, working memory, enjoyment in science lessons and inquiry attitude as co-variates. Also, the two-way factor interaction was taken into account (condition with gender child). Results showed significant main effects of parental pre-knowledge [*T* = 0.18, *F*(6,92) = 2.76, *p* = 0.02, *n*^2^ = 0.15], and children’s inquiry attitude [*T* = 0.19, *F*(6,92) = 2.89, *p* = 0.01, *n^2^* = 0.16] on child talk. No significant interaction effects on child talk were observed.

#### RQ-1

RQ-1: How does parental pre-knowledge affect parent–child interaction during an inquiry activity in the museum?

##### Manipulations

To find out how pre-knowledge affected parents’ and children’s manipulations during inquiry, the five separate univariate ANOVA’s on the outcome variables are reported. Higher amounts of no manipulations (M_0_), *F*(1,91) = 15.63, *p* < 0.001, *n*^2^ = 0.15, and child manipulates alone (M_C_), *F*(1,91) = 9.00, *p* = 0.003, *n*^2^ = 0.09, were observed for parent–child dyads with parental pre-knowledge (see Table 2).

##### Parent talk

To find out how pre-knowledge affected parent talk, the six separate univariate ANOVAs on the outcome variables are reported. Higher amounts of asking open-ended *wh*-questions (C1-P), *F*(1,97) = 32.38, *p* < 0.001, *n*^2^ = 0.25, asking closed questions (C2-P), *F*(1,97) = 14.98, *p* < 0.001, *n*^2^ = 0.13, and lower amount of interpreting results (C4-P), *F*(1,97) = 5.09, *p* = 0.03, *n*^2^ = 0.05, were observed for parents with parental pre-knowledge (see Table 2).

##### Child talk

To find out how pre-knowledge affected child talk, the six separate univariate ANOVAs on the outcome variables are reported. Higher amounts of describing evidence (C3-C), *F*(1,97) = 4.43, *p* = 0.04, *n*^2^ = 0.04, and interpreting results (C4-C), *F*(1,97) = 10.90, *p* = 0.001, *n*^2^ = 0.10, were observed for children of dyads with parental pre-knowledge, compared to children of dyads without parental pre-knowledge.

#### RQ-2

RQ-2: How do person characteristics affect parent–child interaction during an inquiry activity and the possible relation of parental pre-knowledge and parent–child interaction?

##### Manipulations

**FIGURE 2:**
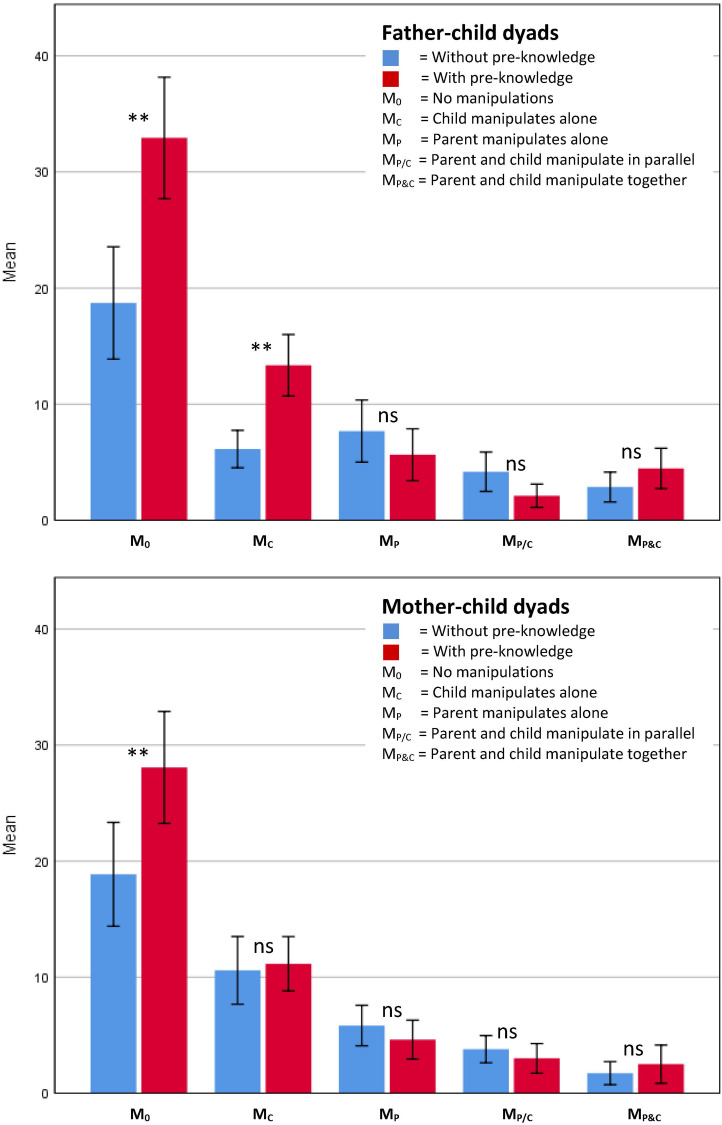
Parental pre-knowledge and parental gender interaction on manipulations. **(A)** Father–child dyads (*N* = 50). **(B)** Mother–child dyads (*N* = 55). Mean values of the five manipulation categories (M_0_, M_C_, M_P_, M_P/C_, and M_P&C_) for the two pre-knowledge conditions (blue = Control condition without parental pre-knowledge, red = Experimental condition with parental pre-knowledge). Error bars = 95% CI. Significances for differences between pre-knowledge conditions are depicted: ^ns^*p* > 0.05; **p ≤ 0.01.

The follow-up univariate ANOVA’s revealed that, compared to parent–son dyads, in parent–daughter dyads, parents more often manipulated alone (MP), *F*(1,91) = 4.20, *p* = 0.04, *n*^2^ = 0.04, and parent–daughter dyads less often manipulated together (M_P&C_), *F*(1,91) = 3.96, *p* = 0.05, *n*^2^ = 0.04. The follow-up univariate ANOVAs also revealed that only for father–child dyads, compared to dyads without parental pre-knowledge, children of dyads with parental pre-knowledge more often manipulated alone (M_C_), *F*(1,91) = 8.30, *p* = 0.005, *n*^2^ = 0.08 (see also Figure 2).

##### Child talk

To investigate how children’s inquiry attitude affects child talk, the six separate univariate ANOVAs on the outcome variables are reported. Children with higher self-reported inquiry attitude more often asked open-ended *wh*-questions (C1-C), *F*(1,97) = 6.10, *p* = 0.02, *n*^2^ = 0.06, and less often asked closed questions (C2-C), *F*(1,97) = 3.96, *p* = 0.05, *n*^2^ = 0.04, compared to children with lower self-reported inquiry attitude (see Table 2).

### Impact of Parental Pre-knowledge on Solution Accuracy

Parents’ and children’s solution accuracy, measured by classifying their drawings in one of four drawing categories, is presented in Table 4. Parental pre-knowledge resulted in higher accuracy for children (χ^2^ = 12.88, *p*_bootstrap_ = 0.003) and parents (χ^2^ = 53.43, *p*_bootstrap_ < 0.001). The most striking difference is that none of the children (0%) in the control condition were able to solve the black-box problem correctly, compared to 15% in the with parental pre-knowledge condition. Looking at fathers and mothers separately, it appears that in the pre-knowledge condition, fathers’ solutions were as good as mothers’ solutions (χ^2^ = 1.443, *p*_bootstrap_ = 0.70). However, in the control condition, fathers gave better solutions than mothers (χ^2^ = 1.443, *p*_bootstrap_ = 0.04).

**TABLE 4:** Parents’ and children’s solution accuracy of the inquiry activity.

		Without pre-knowledge		With pre-knowledge	
		Child	Parent	Child	Parent
K1 (incorrect)	*N* (%)	7 (14)	5 (10)	8 (15)	1 (2)
K2	*N* (%)	10 (20)	2 (4)	17 (31)	5 (9)
K3	*N* (%)	34 (67)	44 (86)	21 (39)	14 (26)
K4 (correct)	*N* (%)	0 (0)	0 (0)	8 (15)	34 (63)

*Total number (*N*) and percentages (%) of parents and children per drawing category (K1, K2, K3, and K4). Column “Without” and “With” are parents (Parent) and children (Child) in the without (*N* = 51) and with (*N* = 54) parental pre-knowledge condition, respectively. K1, incorrect ropes and incorrect connection; K2, correct ropes but incorrect, fixed connection; K3, correct ropes but incorrect, loose connection; and K4, correct ropes and correct loose connection.*

## Discussion

The current study’s main research question concerned the effect of parental pre-knowledge on parent–child interaction during inquiry. We used an experimental design to study this question, and additionally focused on the effects of parents’ and children’s person characteristics on parent–child interactions. Parent–child interactions were assessed by coding non-verbal (manipulations) and verbal behaviors (conversations), and learning was measured as solution accuracy after the parent and child’s inquiry activity. To allow for manipulation of parental pre-knowledge, a black-box was used as the object of inquiry: a closed box where four rope ends stuck out. By inquiring, that is, pulling the ropes, dyads could largely figure out how the ropes were entangled inside the box. Parents in the pre-knowledge condition were shown the entangled ropes in the inside of the box prior to the inquiry activity, without children knowing.

### The Effect of Parental Pre-knowledge on Parent–Child Interaction

Parental pre-knowledge led to differences in parent–child interaction with regard to inquiry time, manipulations, conversations, and learning. Parent and child in the parental pre-knowledge condition, inquired the box for a substantially longer period of time. Below, differences in manipulations, conversations, and learning will be discussed.

#### Manipulations

Children of parents with parental pre-knowledge more often manipulated the ropes on their own than children of parents without pre-knowledge. To the best of our knowledge, research that investigated the relation between parental knowledge and parent–child interaction during inquiry in the museum did not report on children’s individual contribution to manipulations (Palmquist and Crowley, 2007; Benjamin et al., 2010; Eberbach and Crowley, 2017).

A second finding was that parents and children in the parental pre-knowledge condition more often did not manipulate the ropes. It seems that these dyads focused more on verbal exchange than dyads in the condition without parental pre-knowledge. On average, these dyads indeed talked more, during which they did not manipulate. These dyads also inquired for a substantially longer period of time longer (i.e., on average 1 min more) compared to dyads in the without pre-knowledge condition. In line with these findings, Benjamin et al. (2010) report a positive causal effect of parents’ elaborative talk (open-ended *wh*-questions and associations) on dyads’ time spent in the exhibition.

#### Conversations

During the conversations, parents with pre-knowledge less often interpreted results themselves compared to parents without pre-knowledge. Instead, parents with pre-knowledge more often asked questions, both open-ended *wh*-questions and closed questions. That is, results indicate that parents with pre-knowledge behaved more as if they were in the role of being a supporter of the child’s learning process by asking questions, instead of being a learner alongside the child. Especially asking open-ended *wh*-questions is considered to be an important strategy for supporting children’s problem-solving and knowledge seeking behavior in informal (Boland et al., 2003; Eberbach and Crowley, 2017) and formal (Smith and Reiser, 2005) learning context. Children with parents who did have pre-knowledge more often described evidence and interpreted results. Several museum studies demonstrate positive effects of parents’ *wh*-questions on children inquiry and learning (Crowley et al., 2001a; Benjamin et al., 2010; Willard et al., 2019).

Our finding that parental pre-knowledge enhanced asking open-ended *wh*-questions was not evidenced by Benjamin et al. (2010). An explanation for this discrepancy in findings could be that in Benjamin et al.’s (2010) study both parent and child received information, while in the current study, only the parents did, without children knowing. Our results are in line with those of Palmquist and Crowley (2007) who report that parents of novice children talked more and in a more supportive way with their children than parents with expert children. The same relationship is supported by the results of Eberbach and Crowley (2017) who report a correlation of parental pre-knowledge of pollination and the amount of open-ended *wh-*questions asked by parents during a botanical garden visit.

#### Children’s Solution Accuracy

The finding that some of the children in the pre-knowledge condition solved the inquiry problem, compared to none of the children in the without pre-knowledge condition, indicated that parental pre-knowledge facilitated children’s learning. One explanation is that pre-knowledge facilitated parents in scaffolding their children’s learning through inquiry. This explanation is supported by higher amounts of open-ended *wh*-questions that parents in the pre-knowledge condition asked their children. The explanation that parents with pre-knowledge told the inquiry problem’s solution directly to their child is less likely since parents with pre-knowledge less often interpreted results, which in the current study entails giving explanations. A second finding was that, in the control condition, not only the children but also the parents were often unable to solve the inquiry problem in all detail. However, partially correct answers were given by a substantial group of parents and children. That is, by inquiry they could solve important aspects of the task. Nevertheless, this suggests that the task was difficult to solve fully by inquiry alone, that is, without guidance by a parent with pre-knowledge. As the analysis of the conversation shows, the contribution of the parent with pre-knowledge was not explaining the solution, but scaffolding the child in discovering the solution. These results are in line with insights from education effect studies into open-ended inquiry learning (Alfieri et al., 2011) and studies into guided inquiry learning (Van de Pol et al., 2010).

### The Impact of Person Characteristics on Parent–Child Interaction

This study did show ample evidence for the impact of child characteristics on parent–child interaction. Parent characteristics were only in an interaction between gender and parental pre-knowledge related to parent and child’s manipulations.

#### Child Characteristics

The child’s gender was found to be related to how parent and child cooperated during manipulations. Compared to parent–son dyads, in parent–daughter dyads, parents more often manipulated alone and parent and child less often manipulated together during the inquiry activity. Informal science education literature reports on differences in how parents interact with boys and girls while engaging in inquiry activities (Crowley et al., 2001b; Siegel et al., 2007; Luce et al., 2013). As observed in the current study, Siegel et al. (2007) found that, at home and with a closed-ended task, parents behaved more collaboratively with boys than with girls. However, in the same study, in a museum context and with more open-ended activities, parents collaborate more with girls than with boys. Apparently, correlations between the child’s gender and parent–child interaction are highly context and task specific. An explanation for context-related differences in parental behavior with girls and boys, is parents’ gender-biased beliefs about children’s abilities and interests (Sigel and McGillicuddy-De Lisi, 2002; Siegel et al., 2007). In future research, to better understand how the child’s gender relates to parent–child interaction during inquiry, it could be informative to examine the impact of parents’ beliefs about their child’s science achievement and science interest.

Another explanation for the observed differences in cooperation in the current study is that, instead of parents, children acted differently. Whether cooperation is achieved by the child, or is a reaction of the child to the parents’ behavior (or the parent to the child’s behavior) is difficult to disentangle (Tenenbaum and Leaper, 2003). Anecdotally Siegel et al. (2007) observed that boys, more than girls, were taking the lead in performing experiments. In science education literature, the relation between the child’s gender and science achievement has been studied with contradicting results: some studies showed significant differences between boys and girls, while others did not (Sungur and Tekkaya, 2003).

Children’s self-reported inquiry attitude was related to differences in child talk. Children with higher inquiry attitude (e.g., children who prefer to solve a problem by inquiring instead of by being told) more often asked open-ended *wh*-questions, and less often asked closed questions, compared to children with lower inquiry attitude. This relation could be considered as a validation of the TOSRA Inquiry Attitude subscale (Fraser, 1981) in the museum context. Attitude toward science is recognized as an important educational outcome as it relates to lifelong learning (Organisation for Economic Co-operation, and Development [OECD], 2009; Ainley and Ainley, 2011; Sachisthal et al., 2018).

#### Parent Characteristics

Against our expectations, no main effects of parent characteristics on parent–child interaction were observed, besides the experimentally acquired parental pre-knowledge. Museum literature shows that dyads’ time spent in the exhibition was found to be correlated to parents’ educational level (Szechter and Carey, 2009) and parents’ attitude toward science (Tare et al., 2011). Moreover, parent–child interaction was found to be correlated to the parents’ gender (Brown, 1995; Benjamin et al., 2010; Nadelson, 2013; Van Schijndel and Raijmakers, 2016). For example, compared to mothers, fathers tended to be more active when accompanying their child’s exploration of hands-on exhibits (Brown, 1995). Compared to fathers, mothers gave more causal explanations (Van Schijndel and Raijmakers, 2016). However, we did observe an interaction effect of parents’ gender and parental pre-knowledge on manipulations. The interaction effect of parental pre-knowledge and parent gender on manipulations (see also *Section “Conversations”*) revealed that children of father–child dyads, not of mother-child dyads, manipulated more frequently alone in case the parent had pre-knowledge. Relating this result to the accuracy of solutions found by the parents, it seems that fathers without pre-knowledge (relative to fathers with pre-knowledge) were giving children less time to explore alone, because they were finding out the correct solution. Mothers without pre-knowledge did not limit the time of their children to explore alone, resulting in worse solutions than the fathers without pre-knowledge.

### Limitations of the Study

In studying the causal effect of pre-knowledge on parent–child interaction, we purposefully choose a decontextualized inquiry activity (Clough, 2006), to ensure that none of the participants had pre-knowledge about the inquiry activity (i.e., the black-box). Most inquiry exhibits in science museums, however, are contextualized inquiry exhibits based on natural phenomena. One could question therefore, the external validity of the task for science museums practices. After all, black-box activities challenge the acquisition and use of domain-specific knowledge less. However, black-box activities provide similar challenges as encountered when inquiring natural phenomena; they stimulate the use of domain-general strategies as asking questions, experimenting, observing, and interpreting evidence (Lederman and Abd-El-Khalick, 1998). Museum exhibits that foster these inquiry skills, such as APE (Active Prolonged Engagement) exhibits, do not depend strongly on pre-knowledge, in contrast to counterintuitive exhibits (Gutwill, 2008). Our data showed that parents with pre-knowledge did not share their pre-knowledge directly with the child; parents even explained less to their child. Whether this effect of parental pre-knowledge on parent–child interaction will be the same for phenomenon-based inquiry exhibits, we cannot tell. We imagine that having information about “how to solve a specific problem” (black-box) is different from having information about “how a phenomenon works.” In the latter case, the information has a value that goes beyond the situation of the inquiry activity. This “eternity” value could motivate parents to act differently, for example, by sharing this information with the child by interpreting results in addition to asking open-ended *wh*-questions. Also, knowledge about a real-world phenomenon could make parents more interested in the inquiry activity (Tare et al., 2011; Ainley, 2017; Sachisthal et al., 2018) and this could raise new questions that the parents might want to inquire. In future research, it could be informative to further investigate the effect of parental pre-knowledge on parent–child interaction for a series of phenomena, which differ in the extent that they are familiar to the parents.

A second limitation is the generalizability of the outcomes to natural settings. Our test design does not reflect the natural situation of families inquiring at home or during a free-choice science museum visit. The research setting and the presence of the video camera could have motivated participants extrinsically to inquire longer and with more attention, resulting in a longer inquiry time and more interaction between parent and child (Pattison and Shagott, 2015). However, these possible motivating circumstances were the same for both pre-knowledge conditions.

A third limitation is the generalizability of the study outcomes to non-museum settings. Participants were recruited from the museum population and on average were higher educated than the average Dutch. This could have impacted parent–child interaction; however, our data did not show an effect of parental education level on parent–child interaction.

### Implications for Museum Practice

Insights from research about how person characteristics impact family inquiry in the museum are of value for museum practice, for example, when designing for specific audiences (e.g., Dritsas et al., 1998; Dancstep and Sindorf, 2018) or specific learning experiences (e.g., Humphrey and Gutwill, 2005; Gutwill, 2008; Povis and Crowley, 2015). The findings of the current study could be of interest for museum professionals in making informed choices in exhibition design in relation to desired objectives. The current study with the black-box as activity seems especially relevant for exhibits that foster inquiry skills. The current study suggests, for example, that if aiming to support parents in their role of scaffolding the child’s learning, then it may be helpful to opt for phenomena that parents are more familiar with or to provide parents with information about the specific phenomenon. Our results showed that parents with domain-specific pre-knowledge more often scaffolded their child by asking open-ended *wh*-questions. It seems essential that only the parent has this domain-specific pre-knowledge, not the child (cf. Benjamin et al., 2010). Museum research has shown that parental scaffolding behavior can be encouraged by pre-visit instruction (e.g., domain-general process knowledge), for example, an inquiry training (Gutwill and Allen, 2010), an instruction to use elaborative speech (Benjamin et al., 2010), an instruction to explain (Willard et al., 2019), and an instructional video about coaching techniques (Van Schijndel et al., 2010). An interesting aspect of the current study is that, by offering parents a domain-specific knowledge edge (in contrast to scaffolding or inquiry instruction), they spontaneously showed scaffolding behavior by asking more open-ended *wh*-questions without being trained or instructed to do so.

## Conclusion

Children’s science learning is for an important part dependent on how families observe, discuss, and explore science and technology (National Research Council, 2009; Haden, 2010). The current study investigated how person characteristics relate to families’ learning from inquiry activities and demonstrates that parental pre-knowledge affects the way parents interact and explore with their child. Compared to parents without pre-knowledge, parents with pre-knowledge inquired longer, posed more open-ended *wh*-questions and closed questions, and less often interpreted results. The children of parents with pre-knowledge more often manipulated alone, more often described evidence, more often interpreted results, and gave better solutions. In addition, the study demonstrates that child characteristics affected parent–child interaction during inquiry. Boys more often than girls cooperated with their parents, girls more often than boys manipulated alone, and children with a self-reported higher inquiry attitude asked more open-ended *wh*-questions than children with a lower inquiry attitude. By offering parents a knowledge edge, they spontaneously showed scaffolding behavior by asking more open-ended *wh*-questions and they left the interpretation of inquiry results to their children without being trained or instructed to do so. The current study gives an insight into the potential effect of pre-knowledge on parent–child interaction during an inquiry activity and shows that having pre-knowledge can facilitate parents scaffolding behavior and can lead to a different learning situation for both child and parent.

## Data Availability Statement

The datasets generated for this study are available on request to the corresponding author.

## Ethics Statement

The studies involving human participants were reviewed and approved by the Ethics Review Board of the Institute of Education and Child Studies, University of Leiden. Written informed consent to participate in this study was provided by the participants’ legal guardian/next of kin.

## Author Contributions

RF and MR conceived the ideas and analyzed the data. MR designed the study and supervised the data collection. RF wrote the manuscript. TV and MR revised the manuscript. All authors have approved the final manuscript.

## Conflict of Interest

The authors declare that the research was conducted in the absence of any commercial or financial relationships that could be construed as a potential conflict of interest.
